# Transmission of H7N9 influenza virus in mice by different infective routes

**DOI:** 10.1186/1743-422X-11-185

**Published:** 2014-11-03

**Authors:** Linlin Bao, Lili Xu, Hua Zhu, Wei Deng, Ting Chen, Qi Lv, Fengdi Li, Jing Yuan, Yanfeng Xu, Lan Huang, Yanhong Li, Jiangning Liu, Yanfeng Yao, Pin Yu, Honglin Chen, Chuan Qin

**Affiliations:** Institute of Laboratory Animal Sciences, Chinese Academy of Medical Sciences (CAMS) & Comparative Medicine Center, Peking Union Medical Collage (PUMC); Key Laboratory of Human Disease Comparative Medicine, Ministry of Health, No. 5 Pan Jia Yuan Nan Li, 100021 Beijing, Chaoyang District, China; Department of Microbiology and the Research Center of Infection and Immunology, State Key Laboratory for Emerging Infectious Diseases, The University of Hong Kong, 21 Sassoon Road, Pokfulam, Hong Kong, SAR China

**Keywords:** H7N9 influenza virus, Direct contact, Route of transmission, Ocular secretions, Feces, Pharyngeal secretions

## Abstract

**Background:**

On 19 February 2013, the first patient infected with a novel influenza A H7N9 virus from an avian source showed symptoms of sickness. More than 349 laboratory-confirmed cases and 109 deaths have been reported in mainland China since then. Laboratory-confirmed, human-to-human H7N9 virus transmission has not been documented between individuals having close contact; however, this transmission route could not be excluded for three families. To control the spread of the avian influenza H7N9 virus, we must better understand its pathogenesis, transmissibility, and transmission routes in mammals. Studies have shown that this particular virus is transmitted by aerosols among ferrets.

**Methods:**

To study potential transmission routes in animals with direct or close contact to other animals, we investigated these factors in a murine model.

**Results:**

Viable H7N9 avian influenza virus was detected in the upper and lower respiratory tracts, intestine, and brain of model mice. The virus was transmissible between mice in close contact, with a higher concentration of virus found in pharyngeal and ocular secretions, and feces. All these biological materials were contagious for naïve mice.

**Conclusions:**

Our results suggest that the possible transmission routes for the H7N9 influenza virus were through mucosal secretions and feces.

**Electronic supplementary material:**

The online version of this article (doi:10.1186/1743-422X-11-185) contains supplementary material, which is available to authorized users.

## Background

On 19 February 2013, the first patient infected with the novel influenza A H7N9 virus from an avian source showed signs of sickness. More than 347 laboratory-confirmed cases have been reported in mainland China, with 109 cases resulting in death. Most cases of H7N9 infection have occurred in elderly men who had recently been exposed to live poultry. Patients with laboratory-confirmed H7N9 infections had an exposure history that included direct contact with respiratory secretions or fecal material. Exposure to live poultry is a case-fatality risk associated with influenza A H7N9 virus infection [1–6]. Live bird markets are suspected of being the source of human infections; shutting down live poultry markets resulted in an immediate reduction in cases [7]. There is a precedent for the human transmission of H7 influenza, with limited person-to-person transmission observed in an H7N7 outbreak in the Netherlands in 2003 [8]. Three suspected family clusters of cases were reported [9], indicating the possibility of limited human-to-human transmission of the H7N9 virus [10].

Previous studies have reported aerosol transmission of the H7N9 virus among ferrets [11]. Other researchers have used a mouse model to study contact-dependent transmission of influenza A virus [12]. We evaluated the transmission of the H7N9 virus in mice to provide insights into potential human transmissibility.

## Results

### Pathogenicity of influenza virus strains in mice

**Figure 1:**
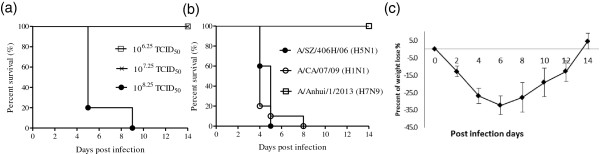
**Avian influenza virus H7N9 (A/Anhui/1/2013) infects mice. (a)** LD_50_ of A/Anhui/1/2013 (H7N9). Representative results of three doses (10^8.25^–10^6.25^ TCID_50_) are shown. **(b)** Survival comparison of mice infected with H5N1, H1N1, or H7N9 viruses. **(c)** Percentage initial weight loss in mice infected with 10^6^ TCID_50_ of H7N9 (A/Anhui/1/2013). Data are presented as the average values from two independent experiments ± SD (*n* =10 per group).

Mice were infected with influenza A/Anhui/1/2013 virus (10^6.25^–10^8.25^ TCID_50_) to determine the LD_50_ and survival of H7N9 in mice (Figure 1a). The LD_50_ values of the three viruses we investigated were 10^7.5^, 10^1^, and 10^4.8^ TCID_50_ for H7N9, H5N1, and H1N1, respectively. At a dose of 10^6^ TCID_50_, mice infected with H5N1 or H1N1 virus died during the observation period (Figure 1b). After inoculation with the H7N9 virus at 10^6^ TCID_50_, weight loss was observed at 2 days post inoculation (dpi; Figure 1c); the average weight loss at 7 dpi was greater than 30%. Mice infected with the H7N9 virus at 10^6^ TCID_50_ exhibited weight loss from 2 dpi but recovered around 8 dpi.

**Figure 2:**
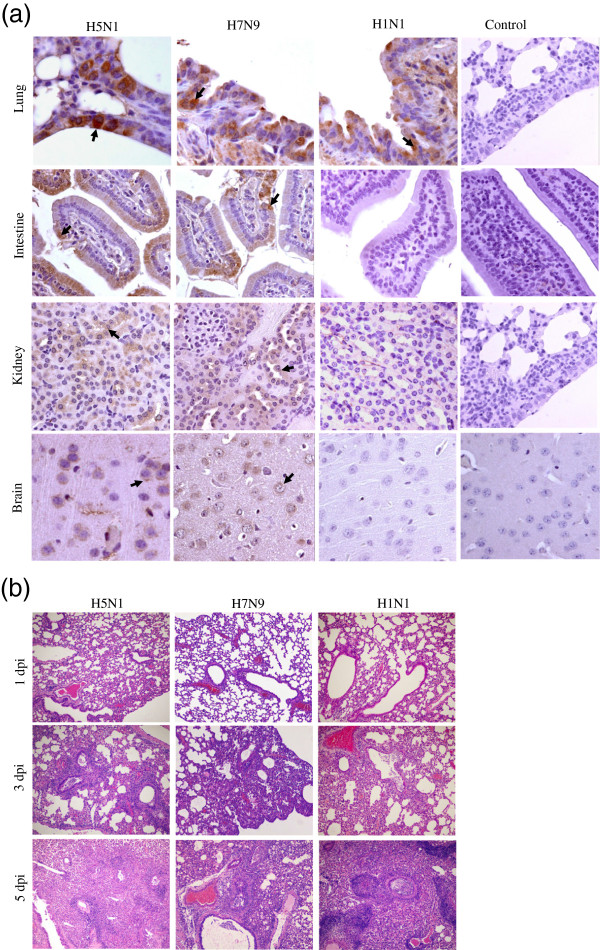
**Histopathology and immunohistochemistry (IHC) analysis of infected mice. (a)** Distribution of H7N9, H5N1, and H1N1 viruses in the tissues of infected mice as determined by IHC. Representative viral antigen distribution in tissues at 3 dpi is shown. Viral antigens are denoted with solid arrows (400× magnification). **(b)** Hematoxylin and eosin stain (HE) staining of lung tissues from infected mice (100× magnification).

Virus titers in the tissues of infected mice administered 10^6^ TCID_50_ of virus were determined using MDCK cells. Viable virus was detected in the lungs of mice infected with any of the influenza viruses from 1–7 dpi (Additional file 1: Table S2). Virus was transiently isolated from the liver, kidneys, and intestines of H5N1- or H7N9-infected mice, but not H1N1-infected mice (Table 1). The virulence of the H7N9 virus was less than that of the H5N1 virus. Viable virus was detected in the brains of 33% (2/6) of H7N9-infected mice at 2 dpi. The distribution of viruses in tissues was assessed using immunohistochemistry incorporating a mouse monoclonal antibody against the influenza HA nucleoprotein (1:200 dilution). Following H7N9 infection viable antigens were primarily located within epithelial cells of the bronchi and alveoli in the lungs, epithelial cells of the intestine, renal tubules in the kidney, and gliocytes in the brain (Figure 2a). H5N1 antigens were also detected in the brain parenchyma (Figure 2a). Viral antigens were mainly detected in the lungs of H1N1-infected mice (Figure 2a). Confluent interstitial pneumonia, necrosis of epithelial cells of the bronchi and alveoli, inflammatory cell infiltration, congestion, and fibrin exudation were observed at 3 and 5 dpi in lung tissues (Figure 2b).

**Table 1:** **Virus titers in the tissues of infected mice**

Virus ^a^	Days post-infection	Virus titer (log _10_TCID _50_ ± SD)							
		Heart	Liver	Spleen	Lung	Kidney	Intestine	Brain	Nose
A/CA/07/09 (H1N1)	5	NP^d^	-^b^	+	4.83 ± 1.7	-	-	-	NP
A/SZ/406H/06 (H5N1)	5	NP	3.62 ± 1.1	1.62 ± 0.23	3.50 ± 1.92	1.17 ± 0.32	1.38 ± 0.27	2.17 ± 0.41	NP
A/Anhui/1/2013 (H7N9)	1	-	-	-	4.00 ± 1.20	-	-	-	3.50 ± 1.38
	2	-	0.81	1.50	5.50 ± 1.90	2.00	-	+^c^	2.50 ± 0.75
	3	-	0.75	-	5.00 ± 1.85	-		-	2.50 ± 0.75
	5	-	-	-	6.00 ± 2.15	-	0.86 ± 0.13	-	3.50 ± 1.38
	7	-	-	-	3.50 ± 1.77	-	0.75	-	2.00 ± 0.38

^a^Mice were infected with influenza viruses at a dose of 10^6^ TCID_50_.

^b^Viable virus was not detected after three passages in MDCK cells.

^c^Cyotpathic effects were observed but TCID_50_ values were less than 0.5.

^d^ *NP*, Not performed.

### H7N9 influenza virus transmission in mice

We sought to determine whether mice infected with the H7N9 influenza virus could transmit virus to naïve mice in close contact. At 24 h post-inoculation (hpi), seven H7N9-naïve mice were introduced into the same cage as three intranasally infected mice (10^7^ TCID_50_). We detected virus in the lungs and intestines of the initially uninfected mice that were placed in contact with mice exposed to H7N9 or H1N1 virus. We were unable to detect H5N1 in either tissue (Table 2).

**Table 2:** **Transmissibility of H7N9 influenza virus in mice put in contact with infected mice**

Virus	Virus titer (mean log _10_TCID _50_ ± SD)									
	Lung		Kidney	Intestine		Brain	Nose	Infected contact mice ^b^	Weight loss (%) ^c^	Seroconverted ^d^
Days post-infection	5	7	5 and 7	5	7	5	5			
A/SZ/406H/06 A/CA/07/09	-		NP^a^	-^f^	-	-	NP	0	0	0
	2.07 ± 0.16	1.47 ± 0.19	NP	-	-	-	+	8/14	11	4/7
A/Anhui/1/2013	1.26 ± 0.41	0.67 ± 0.01	-	1.34 ± 0.25	0.97 ± 0.01	+^e^	+	7/14	6	3/7

^a^ *NP*, Not performed.

^b^Number of contact mice containing virus in the lung, brain, and nose at 5 and 7 days after co-housing.

^c^Maximum proportional weight loss.

^d^HI titers >40.

^e^Cyotpathic effects were observed but TCID_50_ values were less than 0.5.

^f^Viable virus was not detected after three passages in MDCK cells.

**Figure 3:**
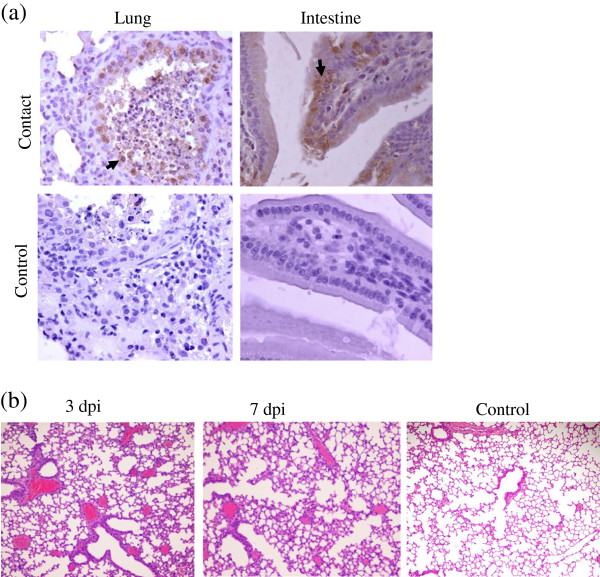
**H7N9 virus is transmissible among mice by direct contact.** Seven naïve mice were placed in direct contact with three mice infected with 10^6^ TCID_50_ of H7N9 virus. **(a)** IHC observations in tissues of mice exposed to H7N9-infected mice. Viral antigen distribution in tissues at 5 days following exposure to infected mice. Viral antigens are denoted with solid arrows (400× magnification). Viral antigens are within the epithelial cells of the bronchi and small intestinal villi, and within infiltrating inflammatory cells of the small intestine. **(b)** HE staining of lung tissue from mice that came in contact with H7N9-infected mice (100× magnification). At 3 days after coming into contact with infected mice, mild dilation of the interstitial pulmonary vasculature was observed in the lungs; congestion was observed after 7 days.

All mice (6/7) exposed to the H7N9 virus exhibited weight loss; the maximal loss observed in one mouse was 6% (Table 2). Around 43% (3/7) of mice exposed to the virus became infected (Table 2). Viable virus was detected in the brain of one mouse and in the nose of another mouse (Table 2). Approximately 6/7 mice exposed to H1N1 exhibited weight loss; the highest level of weight loss approached 11% (Table 2). Of the mice exposed to H1N1, 57% (4/7) became infected (Table 2). In the tissues of the naïve mice that were co-housed with H7N9-infected mice, viral antigens were primarily located in the lung and intestine, similar to their localization in infected mice (Figure 3a). However, virus titers in contact mice were lower than those in the infected mice. Mild lesions were observed in the tissues of naïve mice that were placed in contact with infected mice after 3 days. A dilated area of interstitial pneumonia and congestion were observed in lungs 7 days after contact with infected mice (Figure 3b). We detected antibodies against H7 viruses in seven naïve mice 14 days after contact with infected mice; we also detected H7N9 virus in 3/7 naïve mice (Table 2).

### Secretions from mice infected with H7N9 influenza virus

To evaluate the infectious secretions of H7N9-infected mice, we collected eye and pharyngeal secretions from inoculated mice at 1, 2, and 3 dpi and inoculated them intranasally into mice. Virus was detected in the lungs and intestinal tissues of mice inoculated with the ES-2 inoculum derived from eye secretions, but not with the ES-1 or ES-3 inocula. Virus was detected in the lungs and intestinal tissues of mice administered the TS-1 (throat swab), −2, or −3 inocula. Virus was detected in the lungs and intestines of mice inoculated with feces from H7N9-infected mice (Table 3).

**Table 3:** **Viable virus in naïve mice inoculated with eye, nasal, or fecal samples**

Virus sample type	Virus titer (mean log _10_TCID _50_ ± SD)		
	Lung	Intestine	Nose
ES-1	-	-	-
ES-2	1.17 ± 0.30	0.94 ± 0.62	-
ES-3	-	-	-
TS-1	1.20 ± 0.30	0.94 ± 0.62	-
TS-2	1.02 ± 0.65	0.86	-
TS-3	0.86	0.86	-
F	0.86	0.86	-

### Transmission route of H7N9 in mice

**Figure 4:**
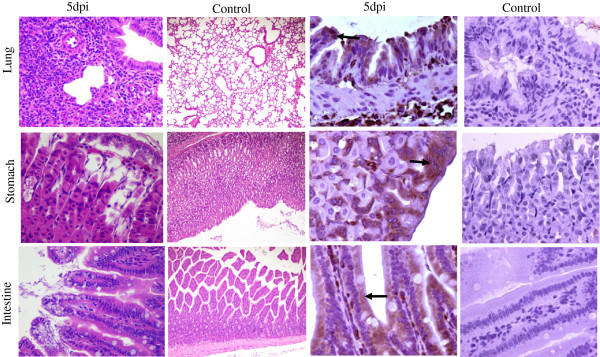
**Transmission of influenza H7N9 virus among mice.** Naïve mice were inoculated with throat secretions collected from infected mice. At 5 dpi, viral antigens were found in epithelial cells of the lungs, gastric and intestinal mucosa, and in infiltrating inflammatory cells of the small intestine (solid arrows; 400× magnification). At 5 dpi, limited interstitial pneumonia and inflammatory cell infiltration, degeneration and necrosis of epithelial cells in the gastric mucosa, and vacuolar degeneration of epithelial cells in small intestine villi was seen (100× magnification).

We tested different transmission routes in contact mice. When using ES inocula, viable virus was detected in the intestines at 2 days, and in both the lungs and intestines at 4 days. Virus antigens were detected in the lungs, stomach, and intestines. TS inoculum was injected into the tail veins of the mice, and viable virus was detected in the same tissues as for the ocular route of administration. Following virus transmission by the fecal-oral route, virus was detected in the nasal passages, trachea, intestines, and lungs; viral antigens were detected in the lungs, stomach, and intestines (Table 4). At 5 dpi, viral antigens were detected in mice infected *via* the oral route, primarily within the epithelial cells of the lungs, gastric mucosa, and intestinal mucosa, and in infiltrating inflammatory cells of the small intestine (Figure 4). At the same time point, we also observed limited interstitial pneumonia and inflammatory cell infiltration, degeneration and necrosis of mucosal and gastric epithelial cells, vacuolar degeneration of the epithelial cells of the villi, and degeneration of the small intestine.

**Table 4:** **Detection of viable virus following inoculation of naïve mice via different routes**

Route of administration	Days	Virus titer in tissue (TCID _50_ ± SD)				Viral antigen distribution in tissues		
		Nose	Trachea	Intestine	Lung	Lung	Stomach	Intestine
Oral	3	-	-	-	-	-	-	-
	5	2.13	2.71	1.80	3.57 ± 1.13	2/3	2/3	2/3
Intravenous	3	-	-	1.80	2.89 ± 0.78	2/3	2/3	2/3
	5	-	-	1.80	-	-	1/3	1/3
Eye	3	-	-	2.05	-	-	1/3	1/3
	5	-	-	1.80	1.80	1/3	1/3	1/3

## Discussion

A second wave of H7N9 viral infections in humans occurred in 2014 across China, with over 50 deaths recorded in 1 month. The Chinese government enforced a temporary closure of live poultry markets in affected provinces, resulting in an immediate reduction in the number of human cases. This provided further evidence of the role live poultry markets play in the spread of the virus, and clearly showed that closure of the markets was an effective control strategy [7]. However, the transmission routes of the H7N9 virus remained unknown, therefore it was still considered a serious threat to human health worldwide [13].

The mouse is a typical model used to evaluate the virulence of influenza viruses, and in its quantitation. It has been reported that a murine model can be used to study contact-dependent transmission of the influenza A virus [12]. In the current study, H7N9 virus-infected mice displayed symptoms of lethality, with pathological changes seen in mice that came in contact with infected mice. We conducted a study on the aerosol transmission of H7N9 in ferrets [11], and used a murine model to study other potential routes of transmission.

Our results indicated that the novel H7N9 virus could efficiently replicate in mice without prior host adaptation. We inoculated BALB/c mice intranasally with A/Anhui/1/2013 to determine H7N9 virus replication, morbidity (as measured by weight loss), and the LD_50_ in mice. The LD_50_ titers for the H7N9 virus were lower than those for H5N1 and H1N1 viruses. Mice inoculated with H7N9 virus showed severe morbidity (30% weight loss), a value comparable to that seen for HPAI H5N1 and H1N1 virus infections, which have a high pathogenicity phenotype in this model. Our findings were similar to those reported in previous studies [14]. The H7N9 virus was detected in extra-respiratory intestines, liver, spleen, kidneys, and brains of infected mice, with viral antigen detected in the same tissues. Weight loss was used as a measurable outcome and a marker of virulence following virus infection in these mice [15–20]. In our study, the duration of H7N9 virus infection in mice was about 12 days, a similar duration to that seen with human H7N9 infections (4–11 days) [15, 21–23]. Virus was detected at higher titers in the lungs than in nasal samples (Table 1). From our transmission experiments we found that virus titers in the lungs of co-housed mice at 5 dpi were higher than those at 7 dpi, with the proportion of infected mice higher than at 7 dpi (Table 2). Results with the H7N9-infected mice were similar to those for H1N1-infected mice. It was previously reported that an avian H7N9 virus effectively replicated in mice with minimal symptoms and that the virus could be transmitted in mice without any pathogenic effects observed [24]. Our results indicate that the H7N9 virus could be transmitted in mice, however weight loss and ruffled coats were observed. The virus was detected at different time points and in several tissues, although virus titer was low.

The transmission of H7N9 influenza virus in ferrets has been shown to occur *via* aerosols and respiratory droplets [11, 14]. Our results show that the H7N9 influenza virus can also be transmitted between mice *via* eye secretions (Table 4). Virus in the lung tissues was detected at 4 dpi following ocular administration of ES inocula in mice.

Eye and pharynx secretions, and feces from infected mice proved to be infectious *via* mucosal, intravenous, and oral routes. Virus was present in lung and intestinal samples (Table 3); at 3 and 7 dpi, mild dilatation of the interstitial pulmonary vasculature was observed in the lungs (Figure 3b). Pathological changes in the lungs were milder in naïve mice than in the infected mice; this difference positively correlated with the amount of virus detected in naïve mice.

Our results indicate that influenza virus can be detected in secretions from the eyes, throat and feces. Virus titer was higher in throat secretions than from eye secretions or fecal samples. Secretions from H7N9-infected mice are able to transmit virus by the ocular, oral, and blood-borne routes. An understanding of viral pathogenesis and of the several transmission routes of the virus would allow for various interventions in animals to prevent a future human pandemic [25].

## Conclusion

Viable virus was found in secretions from the eyes and oral cavities of mice infected with the H7N9 influenza virus. The H7N9 virus was transmitted to contact mice by several routes, including the mucosal and fecal-oral routes. We have provided some leads for interpreting transmission of the novel H7N9 influenza virus in humans.

## Materials and methods

### Viruses

Influenza virus A/Anhui/1/2013 was isolated from a patient with a laboratory-confirmed human A (H7N9) virus infection. The patient was a 35-year-old woman who lived in the Anhui Province of China. On day 6 after the onset of illness, she developed acute respiratory distress syndrome, septic shock, and acute renal damage; the patient died 13 days later [1]. A throat swab (TS) was collected from the patient and propagated in the allantoic sac and amniotic cavity of 9–11-day-old embryonic chicken eggs. The propagated virus was then passaged once in Madin-Darby canine kidney (MDCK) cells. A Q226L (H3 numbering) substitution at the 210-loop of the HA gene was found in this virus. This site has been shown to change receptor binding from avians to humans, possibly increasing the ability of the virus to be transmitted by airborne routes [26]. In addition, the virus encoded PB2 627 K, which is essential for efficient replication of avian influenza viruses in mammals. The highly pathogenic avian influenza A (H5N1) virus A/Shenzhen/406H/06 and 2009 pandemic A (H1N1) virus A/California/07/2009 were also isolated from two patients and propagated in embryonic chicken eggs and MDCK cells. The A (A/Shenzhen/406H/06; H5N1) and 2009 pandemic A (A/California/07/2009; H1N1) viruses were obtained from the University of Hong Kong. MDCK cells were maintained in Eagle’s minimal essential medium (EMEM, Invitrogen) supplemented with 10% fetal bovine serum (FBS), 100 IU/ml penicillin, and 100 μg/ml streptomycin, and were incubated at 37°C/5% CO_2_.

### H7N9 influenza virus in mice

Murine studies were performed in an animal biosafety level 3 (ABSL3) facility using HEPA-filtered isolators. All procedures in this study involving animals were reviewed and approved by the Institutional Animal Care and Use Committee of the Institute of Laboratory Animal Science, Peking Union Medical College (ILAS-PC-2013-006).

The 50% mouse lethal dose (LD_50_) of the virus was determined by intranasally inoculating five groups of mice (*n* =10 mice per group) with 10-fold serial dilutions of the virus in a volume of 50 μl. The LD_50_ was calculated by the method of Reed and Muench [27]. The H5N1 A/Shenzhen/406H/06 and H1N1 A/CA/07/09 strains were used as controls. Mice were infected with 10^6^ TCID_50_ of H7N9, H1N1, or H5N1 virus to compare the virulence of strains.

We anesthetized female 4-week-old mice using isoflurane, and then inoculated them intranasally with the appropriate virus. Heart, liver, spleen, lungs, kidney, intestines, brain tissue specimens and turbinate samples were collected from mice and placed into 10% (w/v) phosphate-buffered saline (PBS) on 1, 2, 3, 5, and 7 dpi (Additional file 2: Table S1). Viruses were titrated in MDCK cells and virus titer was expressed as TCID_50_, as per the Reed-Muench method. We euthanized six mice for every virus at each time point. Additionally, three mice were sacrificed for every virus at each time point, with lungs, intestines, and brain tissues collected for immunohistochemical and pathological analysis. The remaining 10 mice in each group were observed daily for weight loss and mortality until 14 dpi.

### Murine model of H7N9 virus transmission

Three groups of mice were used in this study. For each group, three 4-week-old BALB/c mice were infected, and seven naïve mice were placed in direct contact with infected mice (Additional file 2: Table S1). The three infected mice were lightly anesthetized and inoculated intranasally with 50 μl of 10^7^ TCID_50_ virus in PBS as previously described. At 24 hpi, all materials in the cage were replaced and sterilized, and the seven naïve mice (co-housed mice) were placed in the same cage. The seven co-housed mice were observed daily, with detailed recording of clinical signs and weight loss continuing until 14 dpi. To monitor viral shedding, nasal washes were collected from seven co-housed mice at 3, 5, and 7 dpi. One group of mice was used for each time point. We dripped 50 μl of PBS into the nose of each mouse; the liquid was then allowed to fall back into a collection tube. This was repeated three times. The seven co-housed mice were then euthanized at 3, 5, and 7 dpi. The lungs, kidney, brain, and intestines were collected, and immersed into 1 ml of PBS. Virus titers in the tissues were determined using MDCK cells as previously described [27]. The number of co-housed mice that became infected was calculated by determining the virus titer in the lungs, brain, and nose at 5 and 7 days after co-housing. Pathological examination of the lungs and intestines occurred on days 3 and 7. Sera were obtained from co-housed mice on 14 dpi to confirm seroconversion using hemagglutination inhibition (HI) assays with 0.5% chicken erythrocytes.

### Secretions from H7N9-infected mice

To collect eye secretions (ESs), a swab pre-wetted with PBS was used to wipe the eyes of six mice (5–7 wipes) at 1, 2, and 3 dpi, and then placed into 1 ml of PBS. Throat swabs (TSs) were collected from inoculated mice at 1, 2, and 3 dpi, and transferred to 1 ml of PBS. The ESs and TSs were vortexed and centrifuged (300 g, 10 min), and the supernatants filtered. Five pellets of fecal (F) samples from inoculated animals were collected at 5 dpi and placed into 10% (w/v) PBS, vortexed, and centrifuged (300 g, 10 min). Supernatants were then filtered. The liquid from the ES, TS, and F samples were used to intranasally inoculate mice on days 1, 2, and 3, respectively. Six inoculated mice were then euthanized at 4 dpi (Additional file 2: Table S1). Tissues from the lungs, nasal cavity, and intestines were collected in 1 ml of PBS. Viral titers in tissues were determined using MDCK cells as previously described.

### Transmission route of H7N9 in mice

Conjunctival secretions from H7N9-infected mice were used to wipe the eyes of twelve naïve mice (Eye). Pharynx secretions were injected into the tail veins (150 μl per mouse) of naïve mice (IV). The secretion was concentrated using a hyperfiltration tube (Millipore Amicon). The virus titer of conjunctival secretions was 10^3.8^ TCID_50_, while that for pharynx secretions was 10^3.8^ TCID_50_. Similar virus titers were seen for the fecal suspensions. Six naïve mice were given the fecal suspensions from H7N9-infected by gavage administration (Or; 200 μl per mouse). Six mice infected through Or, Eye and IV routes were autopsied at 3 and 5 dpi. Three mice were used for the isolation of lung, tracheal, and intestinal tissues for pathological and histochemical examinations at 5 dpi. Three mice were used to obtain nasal washes and lung tissues to determine virus titers at 3 and 5 dpi (Additional file 2: Table S1).

### Virus titrations

Virus titrations were performed by end-point titration in MDCK cells. MDCK cells were inoculated with 10^0.5^-fold serial dilutions of homogenized tissues, nasal washes, and TSs. At 1 hpi, cells were washed once with PBS and incubated in 200 μl of infection medium (EMEM, 100 U/ml penicillin, 100 μg/ml streptomycin, and 1 μg/ml TPCK-trypsin). At 3 dpi, supernatants of infected cell cultures were tested for agglutinating activity using turkey erythrocytes as an indicator of cellular infection. Infectious titers were calculated from five replicates using the Reed-Muench method [27].

### Histopathology and immunohistochemistry (IHC)

Animal necropsies were performed according to standard protocols. Samples for histological examination, comprising formalin-inflated lungs, were stored in 10% neutral-buffered formalin, embedded in paraffin, and sectioned (4-μm thickness). The sections were stained with hematoxylin and eosin (HE) for examination by light microscopy, or *via* an immunohistochemical method using a monoclonal antibody against the nucleoprotein of influenza A virus. All slides were examined by a pathologist with 10 years’ experience, and results were confirmed by a second pathologist.

### HI assays

Standard HI assays were performed on post-exposure mouse sera using 0.5% turkey erythrocytes against homologous virus. Sera were collected from inoculated or naïve mice at 14 dpi or 14 days after coming into contact with infected mice, respectively, and tested for the presence of H7N9-specific antibodies.

### Statistical analysis

Differences in body weight, viral copy numbers, and virus titers among groups were analyzed by one-way ANOVA and post-hoc Bonferroni correction. Differences between two groups were analyzed by Student’s *t*-test using SPSS 11.5. A *P-*value less than 0.05 was considered statistically significant.

## Electronic supplementary material

Additional file 1: Table S2: Virus titers in the lungs of infected mice. NP, Not performed. ^b^Days post-infection. ^c^Mice were infected with viruses at a dose of 10^6^ TCID_50._ (DOCX 38 KB)

Additional file 2: Table S1: Experimental mouse groups used in this study. (DOCX 37 KB)
