# Correction to: Transmission of H7N9 influenza virus in mice by different infective routes

**DOI:** 10.1186/s12985-021-01603-2

**Published:** 2021-07-06

**Authors:** Linlin Bao, Lili Xu, Hua Zhu, Wei Deng, Ting Chen, Qi Lv, Fengdi Li, Jing Yuan, Yanfeng Xu, Lan Huang, Yanhong Li, Jiangning Liu, Yanfeng Yao, Pin Yu, Honglin Chen, Chuan Qin

**Affiliations:** 1 Institute of Laboratory Animal Sciences, Chinese Academy of Medical Sciences (CAMS) & Comparative Medicine Center, Peking Union Medical Collage (PUMC); Key Laboratory of Human Disease Comparative Medicine, Ministry of Health, No. 5 Pan Jia Yuan Nan Li, Chaoyang District, Beijing, 100021 China; 2 Department of Microbiology and the Research Center of Infection and Immunology, State Key Laboratory for Emerging Infectious Diseases, The University of Hong Kong, 21 Sassoon Road, Pokfulam, Hong Kong, SAR China

## Correction to: Virology Journal 2014, 11:185 10.1186/1743-422X-11-185

Following publication of the original article [1], the authors realised that in Fig. 2a, the immunohistochemical result of the kidney of the negative control mouse was unfortunately misplaced with lung tissue. The correct photo has now been replaced as shown below. The authors would like to apologise for this mistake and are very sorry for the inconvenience caused.

Authors would like to confirm that this correction does not impact the conclusions of the study.

**Fig. 2:**
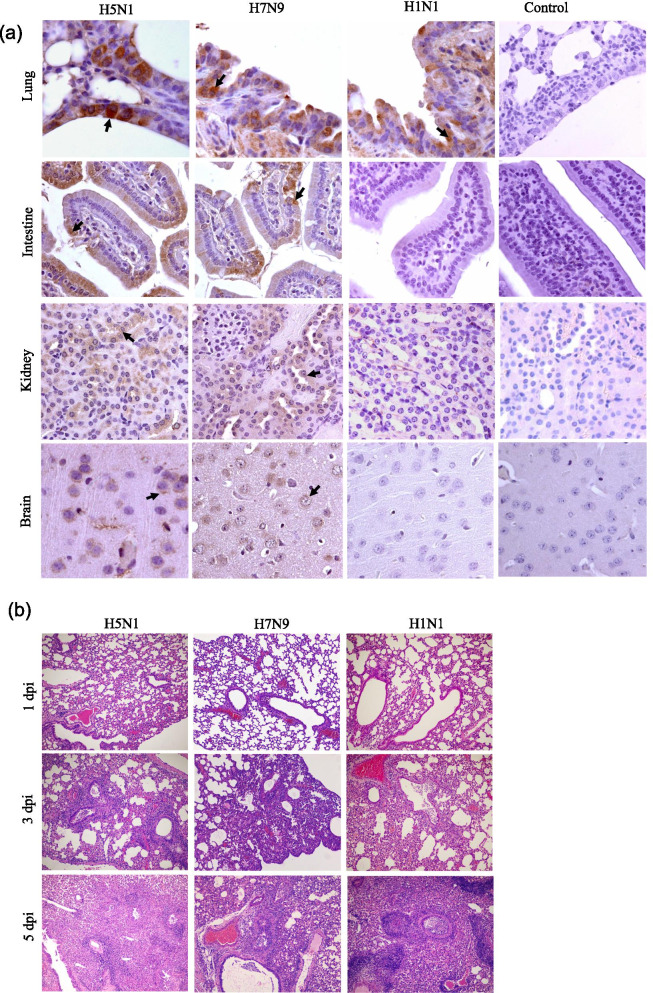
Histopathology and immunohistochemistry (IHC) analysis of infected mice. **a** Distribution of H7N9, H5N1, and H1N1 viruses in the tissues of infected mice as determined by IHC. Representative viral antigen distribution in tissues at 3 dpi is shown. Viral antigens are denoted with solid arrows (×400 magnification). **b** Hematoxylin and eosin stain (HE) staining of lung tissues from infected mice (×100 magnification)
