# Supplementary material for: Transmission of H7N9 influenza virus in mice by different infective routes
Source: Virol J. 2014 Nov 3;11:185. doi: 10.1186/1743-422X-11-185 (PMC4289364; doi:10.1186/1743-422X-11-185)
Supplement: Supplementary file 1 — Additional file 1: Table S2: Virus titers in the lungs of infected mice. NP, Not performed. bDays post-infection. cMice were infected with viruses at a dose of 106 TCID50. (DOCX 38 KB) [file 12985_2013_2512_MOESM1_ESM.docx]

**Additional file 2: Table S2.** Virus titers in the lungs of infected mice.

| Virus | Virus titer (log_10_TCID_50_) | | | | |
| --- | --- | --- | --- | --- | --- |
|  | 1^b^ | 2 | 3 | 5 | 7 |
| A/SZ/406H/06 (H5N1)^c^ | 6.25 | 6.50 | 6.50 | 3.52 | NP |
| A/CA/07/09 (H1N1) | 4.50 | 3.69 | 3.63 | 4.83 | 2.50 |
| A/Anhui/1/2013 (H7N9) | 4.70 | 5.13 | 4.75 | 5.69 | 2.75 |

^a^NP, not performed

^b^Days post-infection

^c^Mice were infected with viruses at a dose of 10^6^ TCID_50_
