# Supplementary material for: Transmission of H7N9 influenza virus in mice by different infective routes
Source: Virol J. 2014 Nov 3;11:185. doi: 10.1186/1743-422X-11-185 (PMC4289364; doi:10.1186/1743-422X-11-185)
Supplement: Supplementary file 2 — Additional file 2: Table S1: Experimental mouse groups used in this study. (DOCX 37 KB) [file 12985_2013_2512_MOESM2_ESM.docx]

**Additional file 1: Table S1.** Experimental mouse groups used in this study.

| Experiment | Inoculum | Inoculation route | Number of mice per test group |
| --- | --- | --- | --- |
| Pathogenicity | H7N9 virus | Intranasal | 10 for weight loss and mortality experiments  30 for virus titration or pathology/IHC |
|  | H5N1 virus |  |  |
|  | H1N1 virus |  |  |
| Transmission | H7N9 virus | Intranasal | 7 for weight loss and seroconversion  21 for virus titration or pathology/IHC |
|  | H5N1 virus |  |  |
|  | H1N1 virus |  |  |
| Secretion | Eye secretions from mice infected with H7N9 virus | Intranasal | 6 for virus titration |
|  | Throat secretions from mice infected with H7N9 virus |  |  |
|  | Fecal samples from mice infected with H7N9 virus |  |  |
| Transmission route | Eye secretions from mice infected with H7N9 virus | Eyes | 12 for virus titration or pathology/IHC |
|  | Throat secretions from mice infected with H7N9 virus | Intravenous |  |
|  | Fecal samples from mice infected with H7N9 virus | Oral |  |
